# Dietary Patterns Associated with Alzheimer’s Disease: Population Based Study

**DOI:** 10.3390/ijerph6041335

**Published:** 2009-04-01

**Authors:** Katarzyna Gustaw-Rothenberg

**Affiliations:** 1 University Memory and Cognition Center, Case Western Reserve Univ. Cleveland, OH, USA; 2 Dept. of Neurodegenerative Diseases IMW, Lublin, Poland; E-Mail: kasiagu@yahoo.ca; Tel.: +1-216-543-4790

**Keywords:** Dietary pattern, factor analysis, Alzheimer’s disease

## Abstract

Recently dietary pattern analysis has emerged as a way for examining diet-disease relations in Alzheimer’s disease. In contrast with the conventional approach, which focuses on a single nutrient or a few nutrients or foods, this method considers overall eating patterns. We examined the dietary patterns defined by factor analysis using data collected with a food-frequency questionnaire in people with Alzheimer’s disease (AD) as compared to healthy controls. The diet data were obtained during population based study of the prevalence of Alzheimer’s disease in a population in Poland. Stratified sampling and random selection strategies were combined to obtain a representative population for screening (age group > 55). From the population screened three times, 71 people were diagnosed with Alzheimer’s according to DSM-IV, and were recruited for further diet risk factors assessment. A group of people with Alzheimer disease (n = 71; F/M 42/29) and the same number of healthy, age and gender matched control were recruited for the study. Patients and their caregivers as well as controls were presented with a food frequency questionnaire based on the 12 food groups. Factor analysis (principal component) was used to derive food patterns. The analysis was conducted using the factor procedure. The factors were rotated by an orthogonal transformation (Varimax rotation) to achieve simpler structure with greater interpretability. Using factor analysis, we identified major eating patterns, one for Alzheimer’s patients and a different one for control group. The AD dietary pattern, FACTOR AD was characterized by a high intake of meat, butter, high-fat dairy products, eggs, and refined sugar, whereas the other pattern, (FACTOR C) was characterized by a high intake of grains and vegetables. These data indicate the existence of dietary patterns defined by factor analysis with data from a food frequency questionnaire, characteristic for Alzheimer’s disease in a Polish population.

## 1. Introduction

Alzheimer's disease (AD) resembles other chronic diseases, whereby a myriad of interconnected factors, including those associated with lifestyle, are involved in disease development [1,2]. Foods, beverages, single food constituents, and unusual eating patterns have been included in several epidemiological risk factor studies [3,4]. Among risk factors oxidative stress and lipid peroxidation may be associated with high fat diets and the pathogenesis of AD [5]. Moreover, dietary antioxidants have been investigated as protection against free radical formation and neurodegenerative disorders [6].Total dietary fat and specific fatty acids have been linked to neurological disorders. High calorie intakes have also been reported to be associated with the development of AD [7]. Recently, dietary pattern analysis has emerged as an approach to examining diet-disease relations in Alzheimer’s disease. In contrast with the conventional approach, which focuses on a single nutrient or a few nutrients or foods, this method considers overall eating patterns [8–11].

Because lifetime dietary patterns as environmental risk factors for Alzheimer's disease (AD) have not been systematically studied, we examined the dietary patterns defined by factor analysis using dietary data collected with a food-frequency questionnaire in people with Alzheimer’s disease as compared to healthy controls.

## 2. Experimental Section

### 2.1. Population Based Sampling Design

The current study is a part of a large population-based study named ***BERCAL*** *(****B****adanie* ***E****pidemiologiczne* ***R****ozpowszechniena* ***C****horoby* ***A****lzheimera i innych form demencji w Województwie* ***L****ubelskim)*.

The participants of the BERCAL study were randomly selected from the population-based sample within the Lublin Region’s 2,182,191.0 inhabitants [12]. The project was carried out to assess the prevalence of dementia and the levels of its risk factors. As a result of the project the prevalence of Alzheimer’s disease in Lublin Region Poland was calculated as 1,634.6/100,000.0 inhabitants.

The BERCAL study design has been described in detail elsewhere [13]. I would like only to mention that dementia was diagnosed by the Diagnostic and Statistical Manual of Mental Disorders 4th edition (DSM-IV) criteria [16] and AD was diagnosed in accordance to the National Institute of Neurological and Communicative Disorders and Stroke/Alzheimer’s Disease and Related Disorders Association (NINCDS-ADRDA) criteria [14].

Normal control subjects were recruited from the same population. Control participants had normal cognition excluding mild cognitive impairment (MCI). The study was carried out in accordance with the local IRB agreement. Written informed consent was obtained from the patient (if possible), the caregiver, and the patient's representative (if applicable) before beginning detailed screening The AD group (n = 71; F/M 42/29) and the same number of healthy, age and gender matched control were recruited to the diet pattern study.

### 2.2. Dietary Pattern Study Design

Patients and their caregivers as well as controls were presented with food frequency questionnaire based on the 12 food groups as described by Szczyglowa [15]. They were asked about their diet earlier in life to make an analysis more reliable in determination diet pattern as a risk factor. Because of the small number of subjects (n = 71) relative to the number of food items, we collapsed the individual food items into 12 predefined food groups.

### 2.3. Statistical Analysis

Factor analysis (principal component) was used to derive food patterns. The analysis was conducted using the factor procedure previously described [8–9]. The factors were rotated by an orthogonal transformation (Varimax rotation) to achieve simpler structure with greater interpretability.

## 3. Results and Discussion

Using factor analysis, we identified major eating patterns - one for Alzheimer’s patients and a different pattern for controls. The first factor-FACTOR AD dietary pattern, was characterized by a high intake of processed meat, butter, high-fat dairy products, eggs, and refined sugar. The other factor, the control pattern, was characterized by a high intake of grains and vegetables.

We conducted a food pattern analysis to describe food consumption patterns associated with risk for Alzheimer’s disease. When food frequencies for cases and controls were analyzed using factor analysis, two major dietary patterns were identified as described before The first factor, FACTOR AD was loaded heavily with, meat, butter and cream as well as different fat, eggs, and refined sugar. The additional characteristics of the FACTOR AD were low amount of fruit and vegetables rich in vitamin C where an amount of beta-carotene wasn’t noticeable at all. The rest of fruit or vegetables groups as well as seeds and legumes where low in this factor as well (Table 1). This factor may be labeled as the low vegetable, high fat and sugar diet pattern. Both AD and C FACTORS were loaded with grain, cereal and bread as well as milk and milk products. The C FACTOR was characterized by a high intake of meat (but no other source of fat) and different kind of vegetables, seeds and legumes. The main difference between FACTORS AD and C was the presence of vegetables rich in beta-carotene. FACTOR C may be labeled as the vegetable, lower fat pattern (Table 2). It can be seen that the high vegetable – low fat pattern, which includes more fruits and vegetables, represents a diet that is similar to those being recommended for all as preventive for cardiovascular diseases, diabetes, and cancer. Since this study provides first known diet pattern noticed in Alzheimer’s patients in the population of Poland it may be a reference for a future more detailed study. For now the advice is to reduce total fat, use plant oils, lower consumption of fat and fat meat, and increase fruits, and vegetables [16].

A large body of evidence shows that free radicals and other oxidative molecules can cause damage that may lead to the development of some cancers, cardiovascular diseases, Parkinson’s disease, and Alzheimer’s disease [6]. This damage can occur over a long period of time, and we are learning that lifelong consumption of fruits and vegetables offers the best protection from oxidative damage [16,17,18].

## 4. Conclusions

These data indicate the existence of dietary patterns defined by factor analysis with data from a food frequency questionnaire, characteristic for Alzheimer’s disease in the population of Poland, especially in Lublin region.

## Figures and Tables

**Table 1.** The Alzheimer’s disease diet pattern. All the food groups listed. For food groups with factor loadings < 0.10, factors were excluded from the table.

**Alzheimer’s Disease**	**FACTOR AD**
1. Grain, Cereals, Bread	0.853
2. Milk and milk products,	0.843
3. Eggs	0.524
4. Meat, Poultry, Fish	0.512
5. Butter and Cream	0.601
6. Fat different than the above	0.385
7. Potatoes	0.373
8. Vegetables and Fruit rich in vitamin C	0.114
9. Vegetables and Fruit rich in beta-carotene	-
10. Vegetables and Fruit different than above	0.112
11. Seeds and Legumes	-
12. Sugar and Sweets	0.573

**Table 2.** The Control diet pattern. All the food groups listed. For food groups with factor loadings < 0.10, factors were excluded from the table.

**Control**	**FACTOR C**
1. Grain, Cereals, Bread	0.715
2. Milk and milk products,	0.888
3. Eggs	0.376
4. Meat, Poultry, Fish	0.703
5. Butter and Cream	0.188
6. Fat different than the above	-
7. Potatoes	0.262
8. Vegetables and Fruit rich in vitamin C	0.338
9. Vegetables and Fruit rich in beta-carotene	0.339
10. Vegetables and Fruit different than above	0.289
11. Seeds and Legumes	0.282
12. Sugar and Sweets	0.244
